# Impact of Holder Pasteurization on Extracellular Vesicles and Immunoregulatory MicroRNAs in Human Breast Milk

**DOI:** 10.1021/acs.jafc.5c11814

**Published:** 2026-01-21

**Authors:** Claudia Gómez Martínez, Luis J. Royo, Sara Escudero Cernuda, Maria Teresa Fernandez-arguelles, Marta Suarez-Rodriguez, Maria Belen Fernandez-Colomer, Maria Luisa Fernandez-Sanchez

**Affiliations:** † Department of Physical and Analytical Chemistry, University of Oviedo, 33006 Oviedo, Spain; ‡ Department of Functional Biology, University of Oviedo, 33006 Oviedo, Spain; § Service of Neonatology, AGC of Childhood and Adolescence, 16474 Hospital Universitario Central de Asturias, 33011 Oviedo, Spain; ∥ Pediatric Research Group, Health Research Institute of the Principality of Asturias (ISPA), 33011 Oviedo, Spain

**Keywords:** breast milk, miRNAs, pasteurization, neonatal health

## Abstract

Human breast milk contains bioactive molecules, including microRNAs (miRNAs) that regulate neonatal development and immunity. Pasteurization ensures microbiological safety in milk banks, but its effects on milk-derived miRNAs remain unclear. This study evaluated the impact of Holder pasteurization (62.5 °C, 30 min) on extracellular vesicle (EVs) morphology and immunoregulatory miRNA expression in exosome, fat, and serum fractions from six donor samples. EVs were characterized by transmission electron microscopy, dynamic light scattering, nanoparticle tracking analysis, and protein quantification. Thirteen miRNAs were analyzed by RT-qPCR. Pasteurization caused morphological alterations in exosomes, reducing particle size and increasing protein concentration, while total particle number remained stable. Expression of eight out of 13 exosomal miRNAs significantly decreased (*p* ≤ 0.05), whereas miRNA profiles in fat and serum fractions were largely preserved.

## Graphical abstract

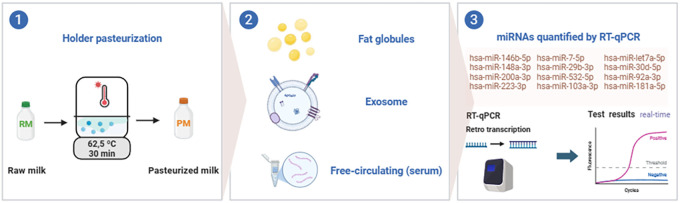

## 1 Introduction

Human breast milk is a complex biological fluid that provides essential nutrients and bioactive compounds crucial for infant growth and development.^1,2^ The bioactive compounds include microRNAs (miRNAs), which are short RNAs that do not encode proteins but instead regulate the expression of other genes important for numerous processes.^2–6^ The miRNAs in mother’s milk are acid-stable and can reach the intestine after ingestion, where they are thought to modulate the infant’s immune system and other processes.^13,14^ For example, miR-146b-5p, miR-181a-5p and miRNAs of the let-7–5p family help regulate innate immune responses, regulate tissue development or control inflammatory responses in neonates.^2,3,16^

The miRNAs in mother’s milk may be present in the lipid fraction (milk fat globules), serum (the aqueous phase obtained by centrifugation after fat separation), and exosomes.^15^ Exosomes are extracellular vesicles (EVs) that enclose RNAs and other biomolecules within a lipid bilayer.^7–12^ Different milk fractions contain different complements of miRNAs,^15,16^ and the complement within each fraction can vary across lactation stage and even over the course of a day.^13,15,16^ These considerations highlight the need to understand the miRNA composition of different fractions of human breast milk and to explore how processing of such milk may alter the miRNA composition.

One such type of processing is pasteurization, typically Holder pasteurization (62.5 °C for 30 min),^20–22^ which is often performed on human breast milk to ensure its microbiological safety during storage in milk banks.^17–20^ While pasteurization is crucial for eliminating pathogenic bacteria, it can reduce levels of beneficial components, such as IgA, alkaline phosphatase, bile salt stimulated lipase, lactoferrin and leptin.^21–25^ It seems likely that pasteurization may affect the miRNA composition of different fractions of milk, especially since industrial processing of milk has been shown to affect the integrity of EVs such as exosomes.^3,26–29^

The present study compared the miRNA composition in exosomes, the fat fraction and serum fraction between raw and pasteurized human breast milk. The findings will contribute to the improvement of processing techniques for keeping milk safe while maintaining the integrity of its bioactive components.

## 2 Materials and Methods

### 2.1 Milk Sample Collection

Human milk samples were collected from six anonymous donor mothers from the Breast Milk Bank of the Central University Hospital of Asturias (HUCA). The study was approved by the Ethics Committee for Research with Medicines of the Principality of Asturias, Spain (approval code: CEImPA 2022.110). Written informed consent was obtained from all participants prior to sample collection, in accordance with the Declaration of Helsinki. As shown in the Table 1, the six samples were collected at different stages of lactation from mothers of both premature and full-term infants. The collected samples were analyzed to provide the diverse profile of lactational stages and compare it between groups. The included women aged 23–46 years, with both caesarean and vaginal deliveries represented. Each milk sample was divided into two fractions, one fraction was stored immediately, and the other was pasteurized. Briefly, milk was heated at constant temperature 62,5 °C for 30 min and cooled down (5 °C) immediately after. Samples were stored at −80 °C until analysis.

**Table 1:** Characteristics of the Six Donors of Mother’s Milk

sample	days postpartum	mother’s age, yr	gestational status at birth	type of delivery	sex of newborn
M1	10	35	term	vaginal	male
M2	26	46	preterm	cesarean	female
M3	11	33	term	cesarean	male
M4	10	44	preterm	cesarean	male
M5	10	24	preterm	vaginal	male
M6	20	23	preterm	cesarean	male

### 2.2 Lipids and Milk Whey Separation

Raw and pasteurized human milk samples, previously stored at −80 °C, were thawed overnight at 4 °C to preserve sample integrity and minimize degradation or microbial growth. The samples were heated in a bath at 37 °C for 15 min and centrifuged for 10 min at 3000*g* at room temperature to separate the milk into three fractions: the upper layer of fat globules, the skim milk and the cellular pellet. The procedure is repeated twice. An additional centrifugation was performed at a higher speed (17,000*g* at 4 °C) than the first three centrifugations to obtain whey as free as possible from fat and cellular debris. The pellet was discarded, and milk whey was transferred into a new tube and used for exosome isolation and RNA extraction. Milk fat was preserved in a single-phase solution of phenol and guanidine thiocyanate (QIAzol), using 1 mL of reagent per gram of fat sample and stored at −80 °C.

### 2.3 Exosomes Isolation

Exosomes were isolated from 10 mL of human milk serum, obtained from both raw and pasteurized milk from each of the 6 samples, using the Plasma/Serum Exosome and Free-Circulating RNA Isolation Maxi Kit (Norgen Biotek Corp., Thorold, ON, Canada) following the manufacturer’s instructions (Figure 1) and stored at −80 °C until further processing.

**Figure 1:**
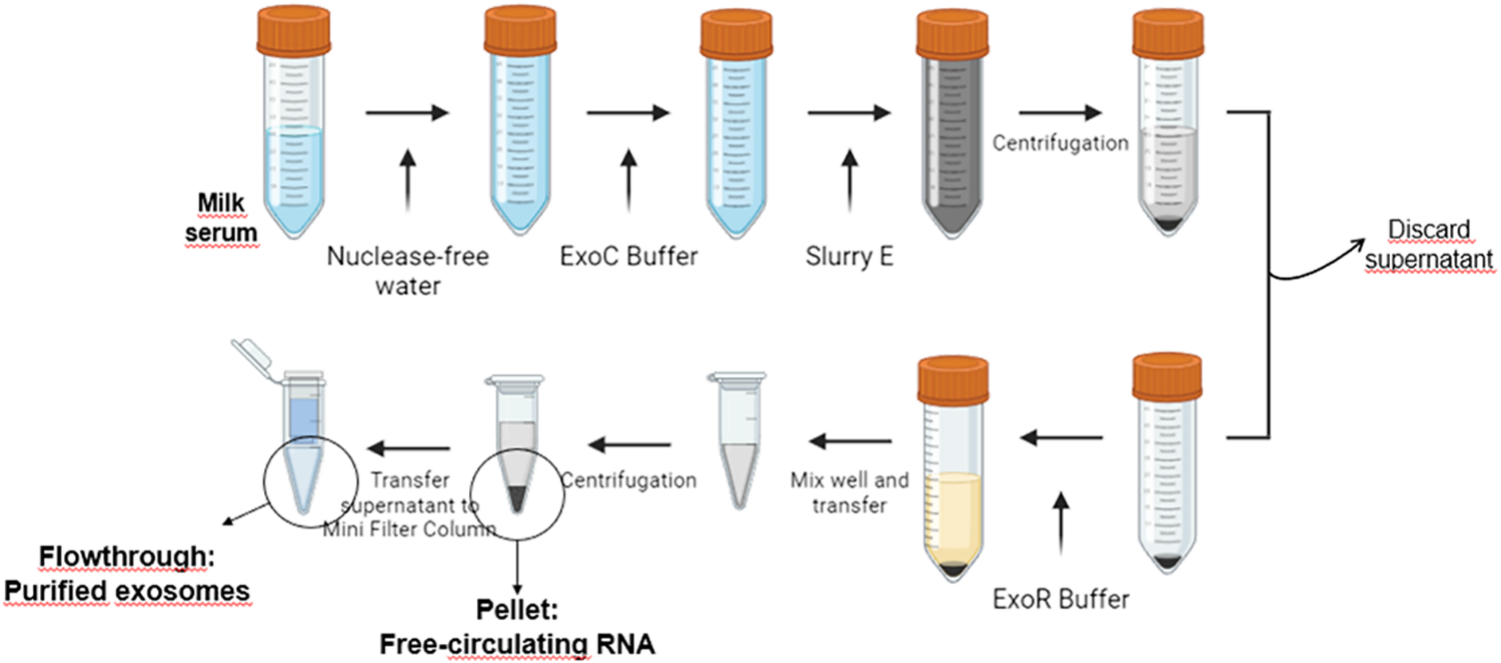
Schematic overview of the experimental procedure used to isolate purified exosomes and free-circulating RNA.

### 2.4 Physical Characterization of Human Milk-Derived Extracellular Vesicles (HMDEVs)

#### 2.4.1 Size, Morphology, and Concentration

For Transmission Electron Microscopy (TEM) analysis, 10 μL aliquots of each sample were fixed using an equal volume of 4% paraformaldehyde solution. The fixed samples were then deposited in Formvar-coated 200–400 mesh copper grids, allowing them to rest for 2 min. Excess solution was carefully removed using filter paper. EVs were stained with a 2% phosphotungstic acid solution for 1 min. Excess stain was removed with filter paper, and the grids were air-dried at room temperature for 10 min. Samples were visualized using a JEM-1011 electron microscope (100 kV, JEOL Ltd., Tokyo, Japan). Images were captured and subsequently processed using ImageJ software (version 1.53c; US National Institutes of Health, Bethesda, MD) to measure the exosome diameters and morphology.

Particle size distribution, polydispersity index (PDI), and ζ-potential of samples of HMDEVs were determined by Dynamic Light Scattering (DLS). The samples were diluted 1:200 in phosphate-buffered saline (PBS), transferred to a disposable polystyrene cuvette and measured a backscatter detection angle of 173° using a Zetasier Nano-ZS ZEN (Malvern Panalytical, Malvern, U.K.). The measurements were performed in triplicate at a constant temperature of 20 °C, using a backscatter detection angle of 173°.

A Nanosight LM10 nanoparticle analyzer (Malvern Panalytical, Malvern, U.K.) equipped with a 405 nm violet laser and a camara level of 15 was also used to estimate the size distribution and concentration of EVs in a solution (particles/mL). Every sample was measured at four different dilutions for interassay studies in order to achieve a concentration within the optimal NTA analysis range (1 × 10^6^ to 1 × 10^9^ particles mL^–1^). Each dilution was captured three times (3 × 30 s acquisition) for the intra-assay assessment. Finally, for sample analysis, a 1:5000 dilution was selected.

#### 2.4.2 HMDEVs Protein Determination

Total protein concentration was determined using the Bicinchoninic Acid Assay (BCA Pierce, Thermo Scientific, Waltham, MA). The calibration graphs between 0 and 60 μg mL^–1^ (Figure S1) were performed with a bovine serum albumin (BSA) standard (Thermo Fisher Scientific) in PBS. For the assay, 10 μL of each standard or sample was processed following the manufacturer’s instructions with minor modifications. The reaction mixture was incubated for 30 min at 55 °C with shaking at 300 rpm. The plate was then allowed to cool to room temperature, and the absorbance was measured at 562 nm using a Multiskan SkyHigh Microplate Spectrophotometer (Thermo Scientific, Waltham, MA).

### 2.5 RNA Isolation

#### 2.5.1 Exosomal and Free-Circulating RNA Isolation

For accurate normalization of the results, 6 fmol of an exogenous miRNA (cel-miR-54-3p) from *Caenorhabditis elegans* (*C. elegans*) was spiked into each sample prior to RNA extraction. Exosomal and Free-Circulating (FC) RNA were isolated from the purified exosomes and the remaining pellet, respectively, using the Plasma/Serum Exosome and Free-Circulating RNA Isolation Maxi Kit (Norgen Biotek Corp., Thorold, ON, Canada) following the manufacturer’s protocols (see Figure 1). The resulting FC and exosomal RNA samples were stored at −80 °C until analysis.

#### 2.5.2 RNA Isolation from Fat

RNA was extracted from the fat layer of human milk samples preserved in QIAzol (see 2.2) using the mirVana miRNA Isolation Kit (Applied Biosystems, Foster City, CA) and following the organic extraction protocol specified by the manufacturer. For normalization, 6 fmol of the exogenous miRNA from *C. elegans* was added to samples prior to RNA extraction. The RNA samples were stored at −80 °C for further analysis.

### 2.6 RT-qPCR Analysis

RNA extracted from the different fractions was used for complementary DNA (cDNA) synthesis using the TaqMan Advanced miRNA cDNA Synthesis Kit (Thermo Fisher Scientific, Waltham, MA), and the cDNA was stored at −20 °C until use. A total of 13 miRNAs present in human breast milk were selected due to their association with infant health and development: hsa-miR-146b-5p, hsa-miR-148a-3p, hsa-miR-200a-3p, hsa-miR-22–3p, hsa-miR-103a-3p, hsa-miR-181a-5p, hsa-miR-223–3p, hsa-miR-29b-3p, hsa-miR-30d-5p, hsa-miR-532–5p, hsa-miR-7–5p, hsa-miR-92a-3p, hsa-miR-let7a-5p. Relative miRNA expression levels were analyzed by reverse transcription quantitative polymerase chain reaction (RT-qPCR) using TaqMan Advanced miRNA Assays (Thermo Fisher Scientific, Waltham, MA). qPCR was performed in a StepOnePlus Real-Time PCR System (Applied Biosystems, Thermo Fisher Scientific) with a 20 μL reaction volume, including 2× TaqMan Fast Advanced Master mix (ThermoFisher Scientific, Waltham, MA), 1 μL of 20× TaqMan Advanced miRNA Assay (ThermoFisher Scientific, Waltham, MA), 4 μL of RNase free water, and 5 μL of 1:10 diluted cDNA. Real time PCR program was set at 95 °C for 20 s, followed by 40 cycles at 95 °C for 1 s and 60 °C for 20 s. All PCR reactions were performed in duplicate, and a maximum of 0.5 threshold cycles (Ct) were permitted between duplicates. Ct values were automatically determined, and relative miRNA expression was quantified using the ΔΔCt method, normalized to exogenous controls.

### 2.7 Statistical Analysis

Differences in relative miRNA levels between pasteurized and raw milk samples were assessed for significance using the Mann–Whitney rank-sum test within SPSS (version 27.1; IBM, Chicago, IL). Differences that were associated with *p* ≤ 0.05 were considered statistically significant.

## 3 Results

### 3.1 Physical Characterization of HMDEVs

#### 3.1.1 Size and Morphology

TEM showed that exosomes isolated from raw milk samples showed the expected intact circular structure with a well-defined lipid bilayer (Figure 2A). Exosomes from pasteurized milk samples, in contrast, showed a deformed structure (Figure 2B). Pasteurization was associated with significantly smaller exosome diameter, based on electron microscopy (120 ± 37 vs 201 ± 62 nm, *p* < 0.0001; Figure 2C) and DLS (197 ± 67 vs 292 ± 103 nm, Figure S2). The range of diameters observed in our preparations of EVs suggested that most were exosomes, though we cannot exclude some contamination with smaller or larger vesicles. Such heterogeneity is common in HMDEVs preparations and may result from both technical and biological factors. The isolation procedure used, based on precipitation and filtration, primarily enriches exosomes but may also coisolate small microvesicles or vesicles derived from cellular debris due to overlapping size ranges.^9,12^ In addition, the complex milk matrix contains casein micelles, milk fat globule membrane fragments, and protein aggregates that can copurify with vesicles despite multiple centrifugation and washing steps. Nonetheless, the observed morphology and size distribution indicate that exosomes represent the predominant vesicular population in the samples.

**Figure 2:**
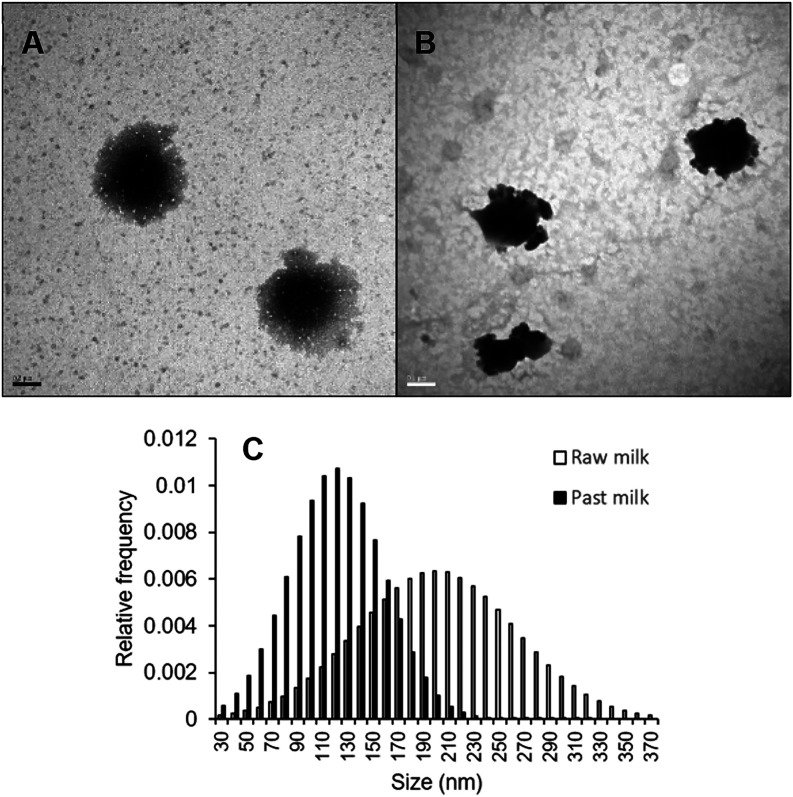
Transmission electron microscopy showing the morphology and approximate size of exosomes. (A) Representative electron micrograph of exosomes from nonpasteurized milk. Scale bar, 200 nm. (B) Representative electron micrograph of exosomes from pasteurized milk. Scale bar, 100 nm. (C) Distribution of exosome diameters in preparations from nonpasteurized milk (open bars) and pasteurized milk (solid bars).

#### 3.1.2 Nanoparticle Tracking Analysis

Nanoparticle tracking analysis (NTA) (Figure 3) revealed a particle concentration of 5.2 ± 0.4 × 10^12^ particles/mL for raw milk-derived EVs and 7.9 ± 0.5 × 10^12^ particles/mL for pasteurized milk-derived EVs.

**Figure 3:**
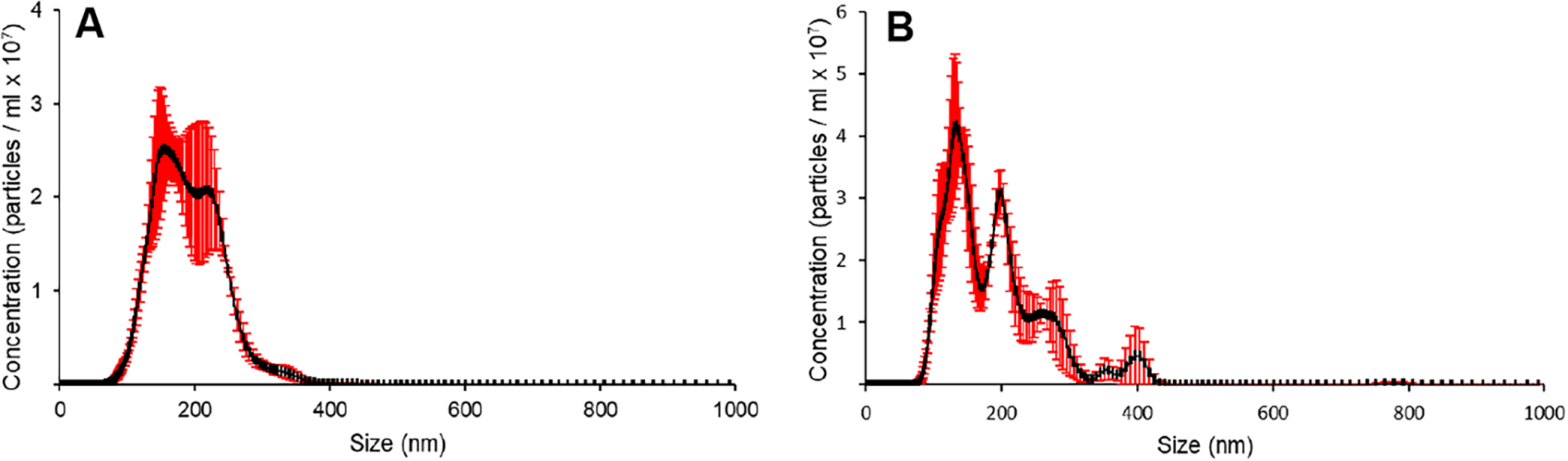
Nanoparticle tracking analysis images of EVs isolated from (A) nonpasteurized and (B) pasteurized human breast milk.

#### 3.1.3 HMDEVs Protein Determination

The protein concentration of the isolated extracellular vesicles, determined by the BCA assay, was 2819 ± 239 μg mL^–1^ for raw milk-derived EVs and 5333 ± 390 μg mL^–1^ for pasteurized human milk derived EVs. These results indicate that vesicles isolated from pasteurized milk exhibit an increased protein yield. Despite the significantly higher protein concentration in pasteurized milk-derived EVs, the difference in particle concentration between the two samples was not as noticeable (Table 2).

**Table 2:** Comparison of Characteristics of Exosome Preparations from Non-Pasteurized and Pasteurized Human Breast Milk

technique	BCA	NTA	DLS		TEM
sample	protein (μg mL^–1^)	concentration (particles/mL)	PDI	size (nm)	size (nm)
raw	2819 ± 239	5.2 ± 0.4 × 10^12^	0.234	292 ± 103	201 ± 62
pasteurized	5333 ± 390	7.9 ± 0.5 × 10^12^	0.164	197 ± 67	120 ± 37

### 3.2 Relative miRNA Level

#### 3.2.1 Exosomal and Free-Circulating RNA

Of the 13 miRNAs in exosomes that were analyzed, levels of the following eight were significantly lower in pasteurized samples than in raw samples (Figure 4): hsa-miR-146b-5p, hsa-miR-148a-3p, hsa-miR-22–3p, hsa-miR-103a-3p, hsa-miR-181a-5p, hsa-miR-29b-3p, hsa-miR-30d-5p, hsa-miR-92a-3p. In contrast, levels of none of the 13 miRNAs in serum (free-circulating RNA) differed significantly between raw and pasteurized milk.

**Figure 4:**
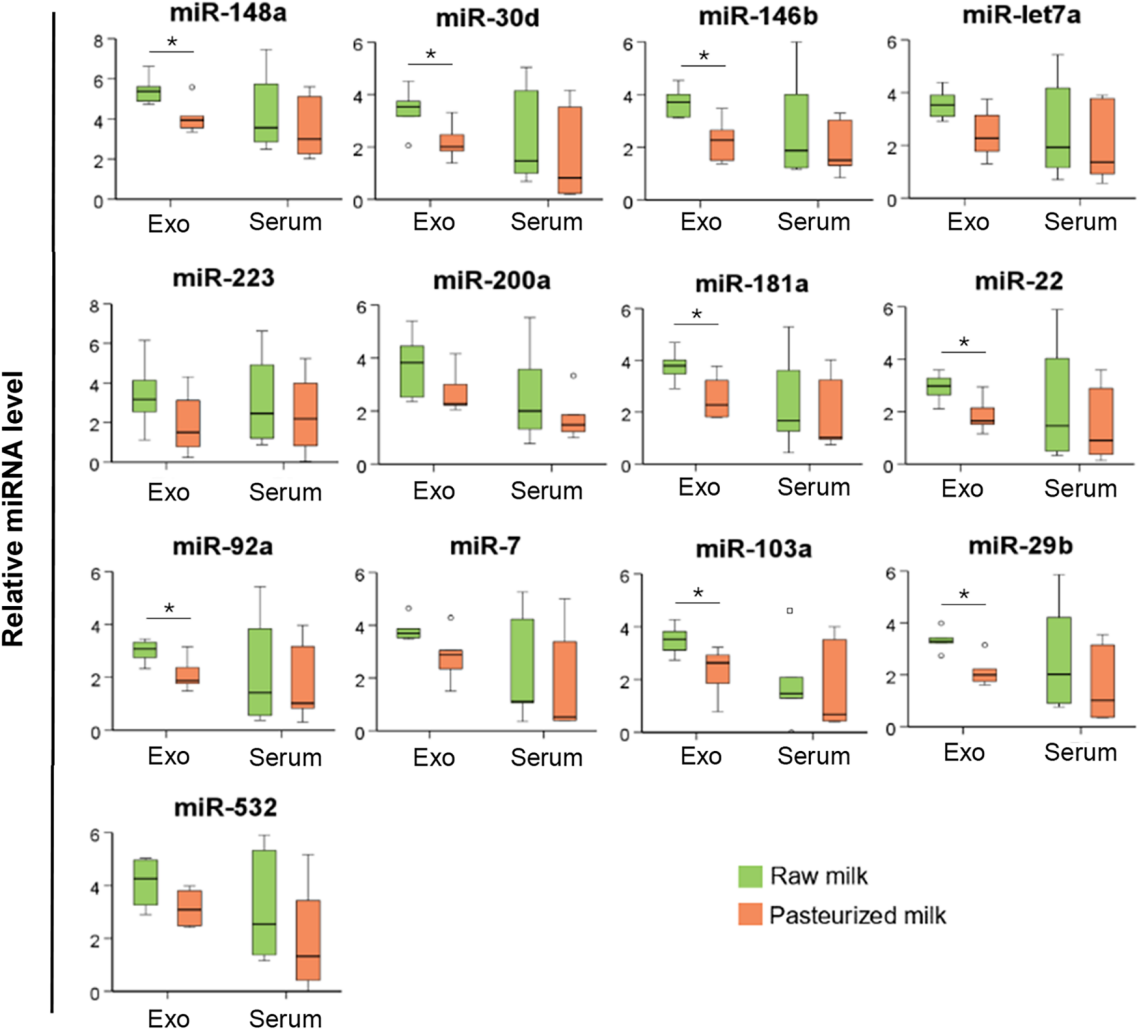
Comparison of relative levels of 13 miRNAs within exosomes (Exo) or in the serum of nonpasteurized (green) or pasteurized (orange) human breast milk. Empty circles indicate mild outliers; empty squares indicate extreme outliers. * *p* ≤ 0.05, based on the Mann–Whitney rank-sum test.

#### 3.2.2 RNA from Fat

Additionally, Figure 5 illustrates the expression levels of miRNAs associated with milk fat in raw and pasteurized milk samples. No statistically significant differences were observed between the two groups.

**Figure 5:**
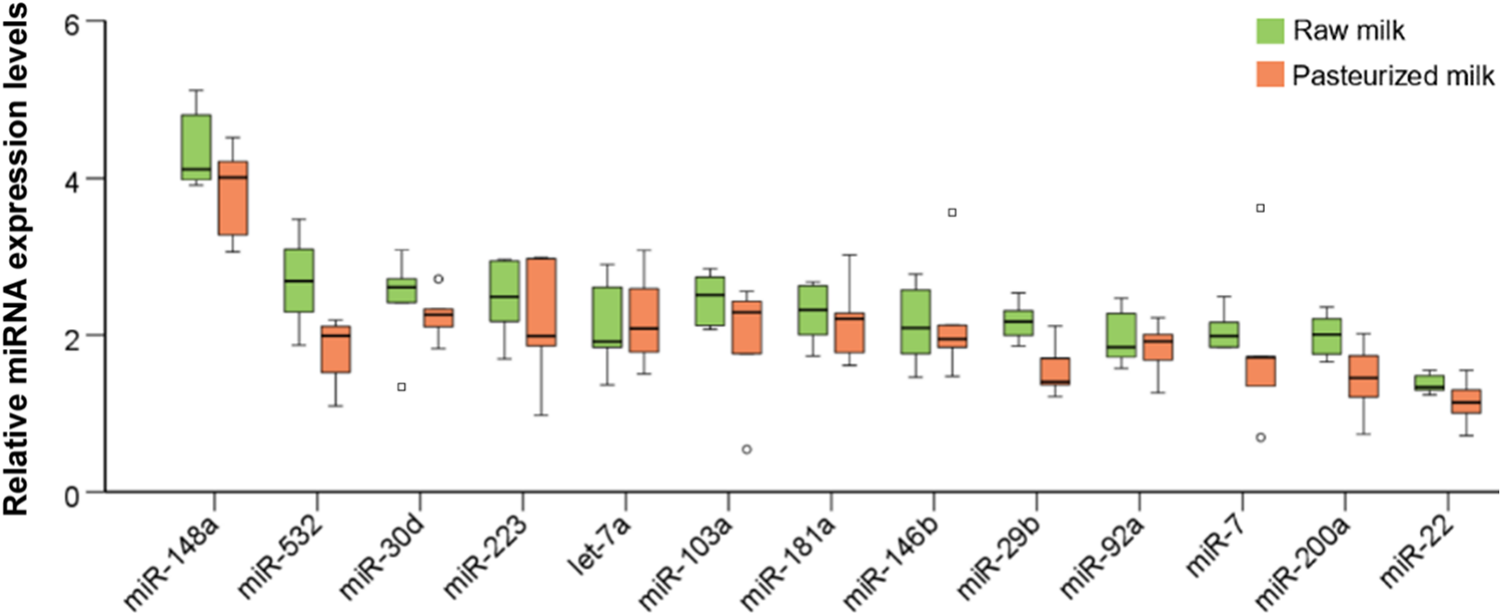
Comparison of relative levels of 13 miRNAs in the fat fraction of nonpasteurized (green) or pasteurized (orange) human breast milk. Empty circles indicate mild outliers; empty squares indicate extreme outliers. * *p* ≤ 0.05, based on the Mann–Whitney rank-sum test.

## 4 Discussion

This study aimed to analyze the effect of pasteurization on the physical characteristics of human milk-derived exosomes and to evaluate the impact of heat treatment on potential differences in the expression profiles of selected immunologically relevant miRNAs involved in newborn development. In addition to assessing the miRNAs encapsulated within exosomes, the expression profiles of miRNAs freely circulating in the milk serum and fat fractions were also analyzed for both types of milk.

Regarding exosome characterization, TEM revealed that exosomes isolated from raw milk exhibited an apparently intact structure, preserving their lipid bilayer. In contrast, exosomes from pasteurized milk showed altered morphology, with evidence of deformation and partial rupture of the membrane and loss surface lipid content.^30^ The partial disruption of the lipid bilayer could reduce the stability of exosomal membranes and alter their ability to fuse with or be internalized by different types of cells. Such structural modifications may compromise the efficiency of miRNA delivery to neonatal epithelial or immune cells. Previous studies have shown that intact exosomal membranes protect miRNAs from enzymatic degradation and facilitate their uptake via endocytosis or receptor-mediated pathways.^8,14^ Therefore, pasteurization might compromise this protective mechanism, leading to altered biodistribution or bioavailability of exosomal cargo. Furthermore, as several of the miRNAs most affected in our study (e.g., miR-146b-5p, miR-181a-5p, miR-148a-3p) are known regulators of immune tolerance and inflammatory signaling,^13,16^ structural damage to exosomes could have downstream effects on immune modulation in the neonate.

The average size observed by TEM in this study (Figure 2) supports the successful isolation of exosomes, as most vesicles fell within the expected size range (30–300 nm). This is consistent with previous reports indicating that exosomes isolated from breast milk are generally larger than EVs derived from other biological fluids.^6,9^

Furthermore, size measurements obtained by both TEM and DLS, indicated slightly larger exosomes in raw milk compared to pasteurized samples. These size differences may reflect the structural damage to the vesicles during pasteurization. The discrepancy in size estimates between TEM and DLS (Table 2) can be attributed to methodological differences: DLS measures the hydrodynamic diameter, which includes the electrical double layer surrounding the vesicles, thus often resulting in an overestimation of particle size compared to TEM.^31^

Protein quantification using the BCA assay revealed a higher total protein content in pasteurized milk compared to raw milk. Although initially unexpected, this finding aligns with the hypothesis of partial membrane disruption in pasteurized exosomes,^21^ leading to increased protein accessibility and, consequently, higher measured concentrations. Since the number of particles per mL did not vary significantly between the two types of milk (Figure 3), no substantial loss in extracellular vesicles concentration was observed. It seems that, pasteurization causes the denaturation of soluble milk proteins, which may lead to their association or adsorption onto the surface of exosomes increasing the protein content associated with the vesicles. This may lead to over estimation of the concentration exosomes based in total protein concentration.^21,23^

Following sample characterization, the expression profiles of 13 selected miRNAs, chosen for their roles in neonatal health, immune development, and gene regulation, were assessed. Significant differences were found between exosomes derived from raw milk and those derived from pasteurized milk in the expression levels of nearly 60% of the miRNAs analyzed (Figure 4). These miRNAs were still detectable in all samples, even at reduced levels in the pasteurized milk, possibly due to leakage or degradation during thermal processing.^3,24^ Previous studies have shown that the miR-29 family is particularly sensitive to thermal treatments,^20^ which aligns with our findings. However, while miR-30d-5p has been reported as relatively stable under High Pressure Processing (HPP),^20^ our results reveal significant differences between raw and pasteurized milk for this marker. Additionally, the significant loss of miR-148a-3p, the most abundant milk miRNA, observed in our study is consistent with other reports investigating heat-based processing methods.^20,24^ No structural features (e.g., secondary structure stability or guanine-cytosine (GC) content) (Table S3) were found among the affected miRNAs to explain their differential sensitivity, despite reports suggesting such factors may influence miRNA stability.^25,33^ Several of the miRNAs most reduced in pasteurized milk exosomes are known regulators of immune and developmental processes in early life. miR-146b-5p and miR-181a-5p modulate innate immune signaling and prevent excessive inflammation in the neonate.^7,13^ miR-148a-3p, one of the most abundant in human milk, promotes oral tolerance and intestinal epithelial maturation by targeting DNA methyltransferases and antigen presentation pathways.^16^ In addition, miR-29b-3p, miR-30d-5p, miR-22–3p, and miR-92a-3p contribute to tissue remodeling and immune homeostasis through the regulation of extracellular matrix components and immune cell differentiation.^2,20^ The reduction of these miRNAs after pasteurization may therefore limit the ability of milk-derived exosomes to deliver immunoregulatory signals to the infant gut, potentially affecting immune maturation and tolerance. However, it remains unknown whether the concentration of these miRNAs in human milk is sufficient to induce measurable gene modulation in the neonate, as suggested by studies performed in bovine milk models.^34^

In the analysis of freely circulating miRNAs in the milk serum, no statistically significant differences were observed between raw and pasteurized simples (Figure 3). However, a consistent trend toward lower expression in the pasteurized samples was noted, accompanied by higher variability among replicates. This may reflect the dynamic and heterogeneous nature of the milk serum, which is more susceptible to external influences and processing-related changes.^15,20,35^

Conversely, in the milk fat fraction (Figure 4), miRNA expression was highly consistent across both milk types, with no significant differences observed. Expression levels were more uniform, which may suggest a protective role of milk fat globules against environmental and processing-related stress, such as temperature variations introduced during pasteurization.^15,32^

Taken together, these findings highlight that while pasteurization does induce certain structural and molecular changes in breast milk exosomes, particularly affecting protein and miRNA content, it does not lead to a complete loss of biologically relevant components. The preservation of key miRNAs and proteins, even at lower levels, suggests that pasteurization remains a valuable method for ensuring the microbiological safety of donor milk without entirely compromising its bioactive potential. Compared to other more aggressive processing methods, such as HPP, high-temperature short-time (HTST), or Ultraviolet–C (UV–C) irradiation, pasteurization appears to strike a more favorable balance between safety and preservation of functionally important milk components.^34^ Nevertheless, recent studies indicate that HPP and HTST treatments can better preserve the structural integrity and biological activity of milk bioactives than Holder pasteurization. For example, HPP-treated milk has been reported to retain higher levels of immunoglobulins, lactoferrin, and intact exosomal membranes, as well as improved stability of immune-related miRNAs such as miR-148a-3p and miR-146b-5p.^20,23,36,37^ However, the large-scale implementation of these alternative technologies in milk banks remains limited due to cost, equipment, and the lack of standardized validation for clinical use. Therefore, Holder pasteurization continues to represent the current reference method, balancing microbiological safety, practicality, and proven clinical outcomes.^19,21^

Despite the valuable insights provided, this study presents certain limitations that must be acknowledged. First, the relatively small number of milk samples analyzed may not fully capture the diversity of human breast milk, and among samples from the same donor could influence the observed results. The samples used were heterogeneous in nature, which may also introduce variability and potentially obscure subtle differences due to the inherent complexity of biological materials. Additionally, although significant differences were found in the expression of several miRNAs, further studies are required to determine the functional relevance of these molecules, particularly to establish whether the loss or reduction of specific miRNAs following pasteurization affects neonatal development or immune regulation. Given the established immunomodulatory functions of several affected miRNAs, their reduced presence in pasteurized milk may have implications for neonatal health. A lower delivery of immune-related miRNAs may limit the contribution of human milk to mucosal immune maturation and tolerance, processes that are especially critical in preterm infants. Such alterations may partly influence susceptibility to inflammatory or immune-mediated conditions, including necrotizing enterocolitis or allergic diseases.^3,7^ Although clinical confirmation is still needed, these observations suggest that preserving the integrity of exosomal miRNAs during milk processing could be relevant to maintaining the protective and developmental benefits of breastfeeding. It also remains unclear whether the affected miRNAs share common biological roles or structural features that make them more susceptible to degradation. Nonetheless, the consistent detection of miRNAs in pasteurized milk, even at lower levels, underscores the potential of pasteurized donor milk to retain important bioactive properties. This support the idea that, when the infant’s own mother’s milk is unavailable, the best alternative is pasteurized donated human milk remains a more beneficial alternative for infant nutrition compared to infant formula or powdered milk products.^38,39^ In fact, one study reported that miR-148a and miR-125b levels in formula were reduced to below 0.2% and 1% of those in mature human milk, respectively, likely due to intensive industrial processing.^40^

In conclusion, this study confirms the presence of exosomes and immunologically relevant microRNAs in both raw and pasteurized human breast milk. Structural analyses revealed that pasteurization induces morphological alterations in exosomes without significantly reducing their cargo. Molecular analyses demonstrated that several miRNAs are present at lower levels in pasteurized samples, yet remain detectable, indicating partial preservation of bioactivity. While the variability inherent to biological samples and the limited sample size represent important limitations, the findings provide a foundation for future research exploring the functional implications of specific miRNA loss due to thermal processing. These results emphasize that pasteurized human milk, although altered, offers important bioactive components absent from infant formula, supporting its use as the preferred alternative for neonates when raw milk is not available.

## Supplementary Material



## Notes

Calibration curve for estimation of total protein using a commercial bicinchoninic acid assay (Figure S1); Distribution of diameters of exosomes isolated from nonpasteurized or pasteurized milk (Figure S2); Sequences and secondary structures of microRNAs analyzed (Table S1) (PDF)

## Abbreviations

BCA: bicinchoninic acid assay
BSA: bovine serum albumin
cDNA: complementary DNA
*C. elegans*: *Caenorhabditis elegans*
Ct: threshold cycle
DLS: dynamic light scattering
EVs: extracellular vesicles
Exo: exosome
FC: free-circulating
GC: guanine-cytosine
HMDEVs: human milk-derived extracellular vesicles
HPP: high pressure processing
HTST: high-temperature short-time
miRNAs: microRNAs
NTA: nanoparticle tracking analysis
PBS: phosphate-buffered saline
PDI: polydispersity index
RT-qPCR: reverse transcription quantitative polymerase chain reaction
TEM: transmission electron microscopy
UV–C: ultraviolet-C

## References

[1] Cintio M., Polacchini G., Scarsella E., Montanari T., Stefanon B., Colitti M. (2020). Microrna milk exosomes: From cellular
regulator to genomic marker. Animals.

[2] Kim K. U., Han K., Kim J., Kwon D. H., Ji Y. W., Yi D. Y., Min H. (2023). The protective
role of exosome-derived MicroRNAs and proteins from
human breast milk against infectious agents. Metabolites.

[3] Lamberti M. F. T., Parker L. A., Gonzalez C. F., Lorca G. L. (2023). Pasteurization of
human milk affects the miRNA cargo of EVs decreasing its immunomodulatory
activity. Sci. Rep..

[4] Freiría-Martínez L., Iglesias-Martínez-Almeida M., Rodríguez-Jamardo C., Rivera-Baltanás T., Comís-Tuche M., Rodrígues-Amorím D., Fernández-Palleiro P. (2023). Human breast milk microRNAs, potential players in the regulation
of nervous system. Nutrients.

[5] Yun B., Kim Y., Park D. J., Oh S. (2021). Comparative analysis of dietary exosome-derived
microRNAs from human, bovine and caprine colostrum and mature milk. J. Anim. Sci. Technol..

[6] Słyk-Gulewska P., Kondracka A., Kwaśniewska A. (2023). MicroRNA as a new bioactive component
in breast milk. Non-coding RNA Res..

[7] Carr L. E., Virmani M. D., Rosa F., Munblit D., Matazel K. S., Elolimy A. A., Yeruva L. (2021). Role of human
milk bioactives on
infants’ gut and immune health. Front.
Immunol..

[8] Lässer C., Alikhani V. S., Ekström K., Eldh M., Paredes P. T., Bossios A., Sjöstrand M. (2011). Human saliva, plasma
and breast milk exosomes contain RNA: uptake by macrophages. J. Transl. Med..

[9] Hu Y., Thaler J., Nieuwland R. (2021). Extracellular vesicles in human milk. Pharmaceuticals.

[10] Zempleni J., Sukreet S., Zhou F., Wu D., Mutai E. (2019). Milk-derived
exosomes and metabolic regulation. Annu. Rev.
Anim. Biosci..

[11] O’Reilly D., Dorodnykh D., Avdeenko N. V., Nekliudov N. A., Garssen J., Elolimy A. A., Petrou L. (2021). Perspective:
the role of human breast-milk extracellular vesicles in child health
and disease. Adv. Nutr..

[12] Jiang X., You L., Zhang Z., Cui X., Zhong H., Sun X., Ji C., Chi X. (2021). Biological
properties of milk-derived extracellular
vesicles and their physiological functions in infant. Front. Cell Dev. Biol..

[13] Kosaka N., Izumi H., Sekine K., Ochiya T. (2010). microRNA as a new immune-regulatory
agent in breast milk. Silence.

[14] Kahn S., Liao Y., Du X., Xu W., Li J., Lönnerdal B. (2018). Exosomal microRNAs in milk from mothers
delivering
preterm infants survive in vitro digestion and are taken up by human
intestinal cells. Mol. Nutr. Food Res..

[15] Li R., Dudemaine P. L., Zhao X., Lei C., Ibeagha-Awemu E. M. (2016). Comparative
analysis of the miRNome of bovine milk fat, whey and cells. PLoS One.

[16] Ahlberg E., Al-Kaabawi A., Thune R., Simpson M. R., Pedersen S. A., Cione E., Jenmalm M. C., Tingö L. (2023). Breast milk
microRNAs: Potential players in oral tolerance development. Front. Immunol..

[17] Salazar S., Chávez M., Delgado X., Eudis Rubio T. P. (2009). Lactancia
Materna. Arch. Venez. Pueric. Pediatr..

[18] Luna M. S., Martin S. C., Gómez-de-Orgaz C. S. (2021). Human milk
bank
and personalized nutrition in the NICU: a narrative review. Eur. J. Pediatr..

[19] Moro G. E., Billeaud C., Rachel B., Calvo J., Cavallarin L., Christen L., Escuder-Vieco D. (2019). Processing of donor
human milk: update and recommendations from the European Milk Bank
Association (EMBA). Front. Pediatr..

[20] Smyczynska U., Bartlomiejczyk M. A., Stanczak M. M., Sztromwasser P., Wesolowska A., Barbarska O., Pawlikowska E., Fendler W. (2020). Impact of processing method on donated human breast
milk microRNA content. PLoS One.

[21] Peila C., Moro G., Bertino E., Cavallarin L., Giribaldi M., Giuliani F., Cresi F., Coscia A. (2016). The effect
of holder pasteurization on nutrients and biologically-active components
in donor human milk: a review. Nutrients.

[22] dos
Santos B. G., Perrin M. T. (2022). What is known about human milk bank
donors around the world: a systematic scoping review. Public Health Nutr..

[23] Kleinjan M., Van Herwijnen M. J., Libregts S. F., van Neerven R. J., Feitsma A. L., Wauben M. H. (2021). Regular industrial processing of
bovine milk impacts the integrity and molecular composition of extracellular
vesicles. J. Nutr..

[24] Howard K. M., Kusuma R. J., Baier S. R., Friemel T., Markham L., Vanamala J., Zempleni J. (2015). Loss of miRNAs
during processing
and storage of cow’s (Bos taurus) milk. J. Agric. Food Chem..

[25] el
Qassim L. A., Martinez B., Rodríguez A., Davalos A., de las Hazas M. C. L., Miranda M. M., Royo L. J. (2023). Effects
of cow’s milk processing on microRNA levels. Foods.

[26] Carneiro L., Marousez L., Van Hul M., Tran L. C., De Lamballerie M., Ley D., Cani P. D. (2023). The sterilization of human milk by holder
pasteurization or by high hydrostatic pressure processing leads to
differential intestinal effects in mice. Nutrients.

[27] Kontopodi E., Stahl B., van Goudoever J. B., Boeren S., Timmermans R. A., den Besten H. M., Van Elburg R. M., Hettinga K. (2022). Effects of high-pressure
processing, UV-C irradiation and thermoultrasonication on donor human
milk safety and quality. Front. Pediatr..

[28] Gayà A., Calvo J. (2018). Improving pasteurization to preserve
the biological components of
donated human milk. Front. Pediatr..

[29] Hansen M. S., Gregersen S. B., Rasmussen J. T. (2022). Bovine milk processing impacts characteristics
of extracellular vesicle isolates obtained by size-exclusion chromatography. Int. Dairy J..

[30] Zhu L., Fu S., Li L., Liu Y. (2022). Changes of extracellular
vesicles
in goat milk treated with different methods. LWT.

[31] Stetefeld J., McKenna S. A., Patel T. R. (2016). Dynamic
light scattering: a practical
guide and applications in biomedical sciences. Biophys. Rev..

[32] Golan-Gerstl R., Shiff Y. E., Moshayoff V., Schecter D., Leshkowitz D., Reif S. (2017). Characterization and
biological function of milk-derived miRNAs. Mol. Nutr. Food Res..

[33] Yang J., Elbaz-Younes I., Primo C., Murungi D., Hirschi K. D. (2018). Intestinal
permeability, digestive stability and oral bioavailability of dietary
small RNAs. Sci. Rep..

[34] de
las Hazas M.-C. L., Del Pozo-Acebo L., Hansen M. S., Gil-Zamorano J., Mantilla-Escalante D. C., Gómez-Coronado D., Marín F., Garcia-Ruiz A., Rasmussen J. T., Dávalos A. (2022). Dietary bovine milk miRNAs transported in extracellular
vesicles are partially stable during GI digestion, are bioavailable
and reach target tissues but need a minimum dose to impact on gene
expression. Eur. J. Nutr..

[35] Zhang Y., Xu Q., Hou J., Huang G., Zhao S., Zheng N., Wang J. (2022). Loss of bioactive
microRNAs in cow’s milk by ultra-high-temperature
treatment but not by pasteurization treatment. J. Sci. Food Agric..

[36] Pitino M. A., O’Connor D. L., Unger S., Kim B. J., Doyen A., Wazed M. A., Kumar S., Pouliot Y., Stone D., Dallas D. C. (2025). Comparative
proteomic analysis of donor human milk
treated by high-pressure processing or Holder pasteurization on undigested
proteins across dynamic simulated preterm infant digestion. Food Chem..

[37] Liang N., Mohamed H. M., Kim B. J., Burroughs S., Lowder A., Waite-Cusic J., Dallas D. C. (2023). High-Pressure Processing
of Human Milk: A Balance between Microbial Inactivation and Bioactive
Protein Preservation. J. Nutr..

[38] Fan Y., Li Z., Hou Y., Tan C., Xiong S., Zhong J., Xie Q. (2024). Effects of
Different Processing on miRNA and Protein in Small Extracellular
Vesicles of Goat Dairy Products. Nutrients.

[39] Liu Y., Zhang W., Han B., Zhang L., Zhou P. (2020). Changes in
bioactive milk serum proteins during milk powder processing. Food Chem..

[40] Chiba T., Kooka A., Kowatari K., Yoshizawa M., Chiba N., Takaguri A., Fukushi Y. (2022). Expression
profiles of hsa-miR-148a-3p and hsa-miR-125b-5p in human breast milk
and infant formulae. Int. Breastfeed. J..

