# Supplementary material for: Impact of Holder Pasteurization on Extracellular Vesicles and Immunoregulatory MicroRNAs in Human Breast Milk
Source: J Agric Food Chem. 2026 Jan 21;74(4):3616–23. doi: 10.1021/acs.jafc.5c11814 (PMC12879930; doi:10.1021/acs.jafc.5c11814)
Supplement: Supplementary file 1 [file jf5c11814_si_001.pdf]

Supplementary Information

Impact of Holder Pasteurization on Extracellular Vesicles and Immunoregulatory microRNAs in Human Breast Milk

Claudia Gómez Martínez[a,d], Luis J. Royo[b], Sara Escudero Cernuda[a], Maria Teresa Fernandez-arguelles[a], Marta Suarez-Rodriguez[c], Maria Belen Fernandez-Colomer[c,d] and Maria Luisa Fernandez-Sanchez[a,d*]

[a] *Department of Physical and Analytical Chemistry, University of Oviedo, 33006 Oviedo, Spain*

[b] *Department of Functional Biology, University of Oviedo, 33006 Oviedo, Spain*

[c] *Service of Neonatology, AGC of childhood and adolescence, Hospital Universitario Central de Asturias, 33011 Oviedo, Spain*

[d] *Pediatric Research Group, Health Research Institute of the Principality of Asturias (ISPA), 33011 Oviedo, Spain*

Corresponding authors:

María Luisa Fernández-Sánchez

Department of Physical and Analytical Chemistry, University of Oviedo.

Avda. Julián Clavería, 8, 33006 Oviedo, Asturias, Spain.

e-mail: marisafs@uniovi.es. Tel.: +34 985 10 3071

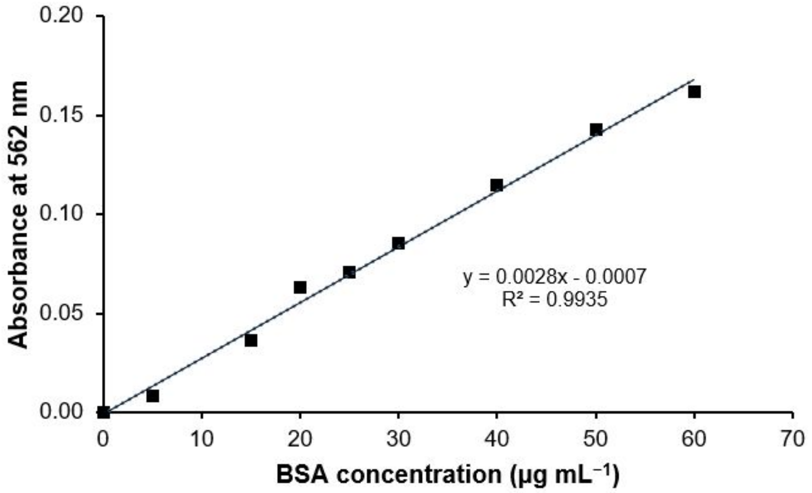

Figure S1. Calibration curve based on bovine serum albumin (BSA) for estimation of total protein in breast milk exosomes using a commercial bicinchoninic acid assay.

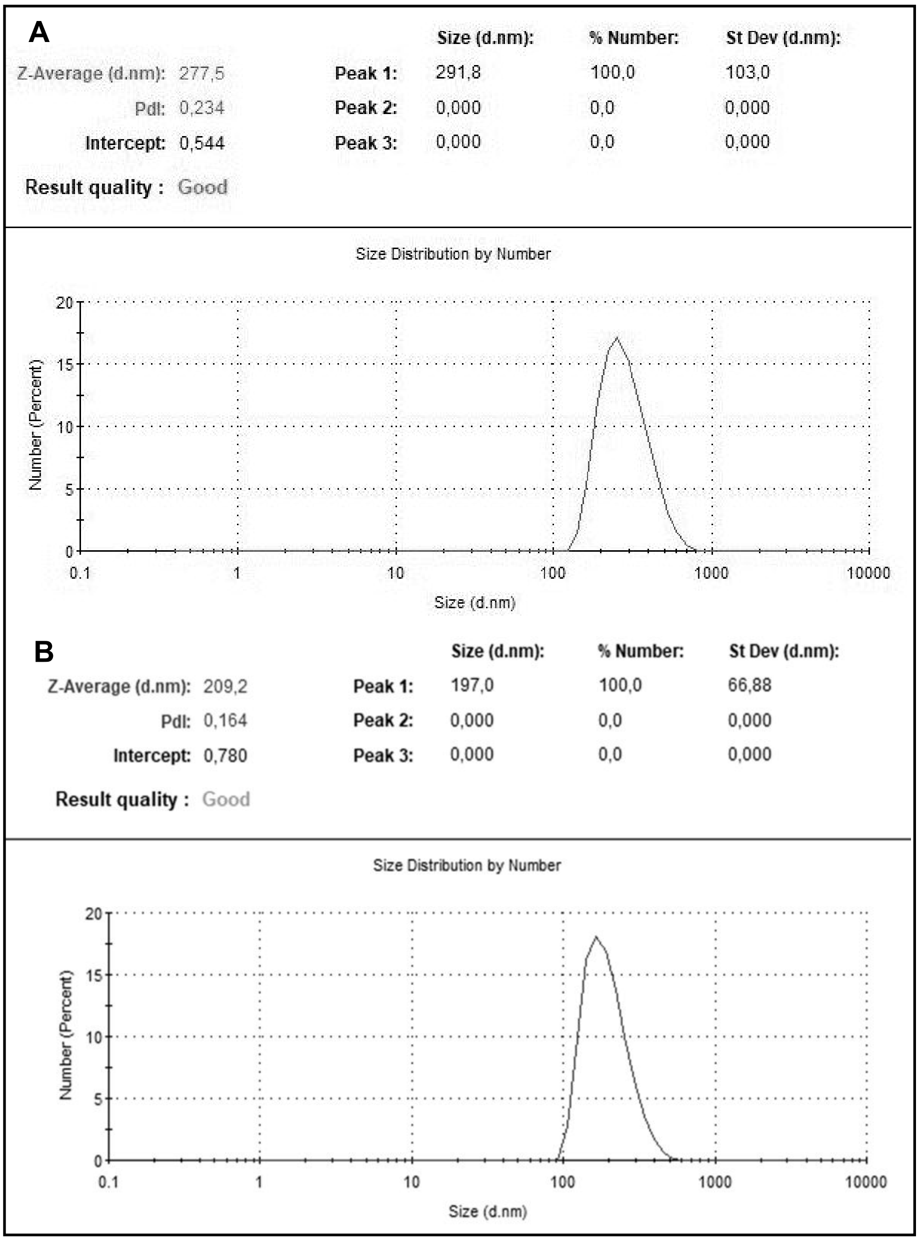

Figure S2. Distribution of diameters of exosomes isolated from **(A)** non-pasteurized or **(B)** pasteurized milk. The respective major peaks showed diameters of 291.8 nm (polydispersity index of 0.234) and 197.0 nm (polydispersity index of 0.164).

Table S1. Sequences and secondary structures of microRNAs analyzed in milk fractions in this study

miRNA	Sequence *	Secondary structure **
hsa-miR-146b-5p	UGAGAACUGAAUUCCAUAGGCUG	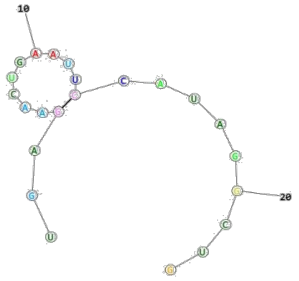
hsa-miR-148a-3p	UCAGUGCACUACAGAACUUUGU	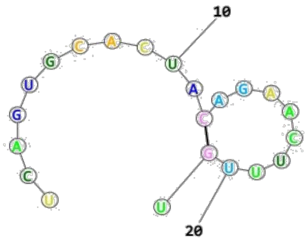
hsa-miR-200a-3p	UAACACUGUCUGGUAACGAUGU	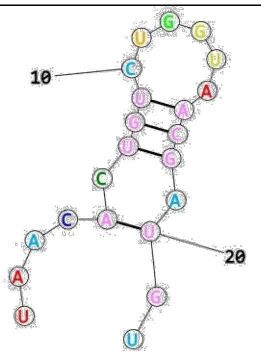
hsa-miR-22-3p	AAGCUGCCAGUUGAAGAACUGU	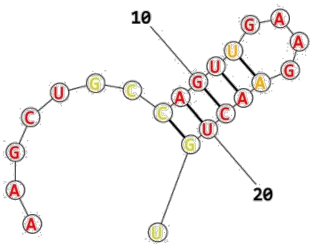

hsa-miR-103a-3p	AGCAGCAUUGUACAGGGCUAUGA	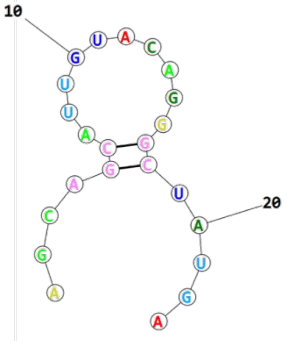
hsa-miR-181a-5p	AACAUUCAACGCUGUCGGUGAGU	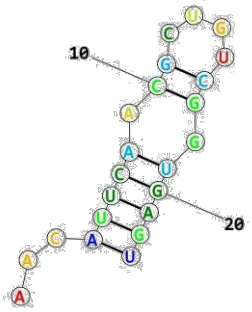
hsa-miR-223-3p	UGUCAGUUUGUCAAAUACCCCA	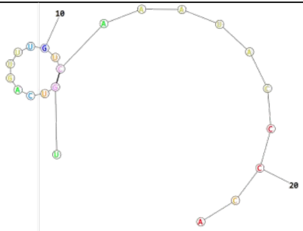
hsa-miR-29b-3p	UAGCACCAUUUGAAAUCAGUGUU	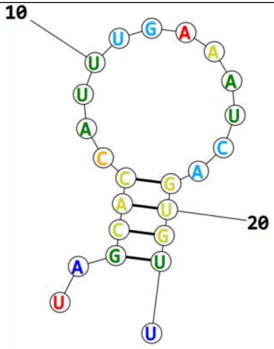
hsa-miR-30d-5p	UGUAAACAUCCCCGACUGGAAG	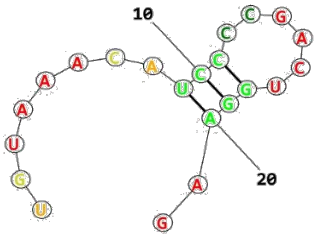

hsa-miR-532-5p	CAUGCCUUGAGUGUAGGACCGU	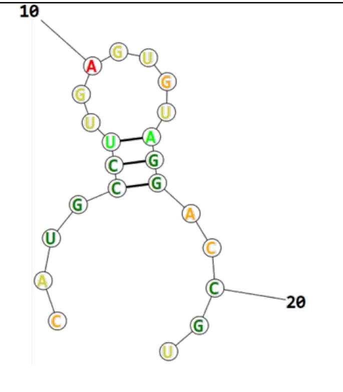
hsa-miR-7-5p	UGGAAGACUAGUGAUUUUGUUGUU	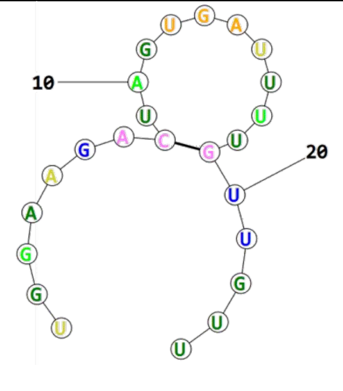
hsa-miR-92a-3p	UAUUGCACUUGUCCCGGCCUGU	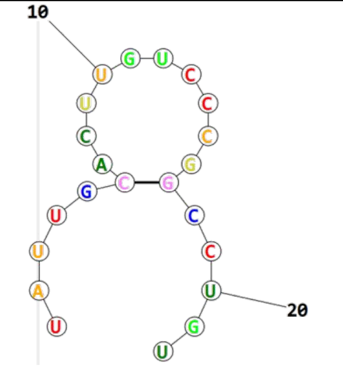
hsa-miR-let7a-5p	UGAGGUAGUAGGUUGUAUAGUU	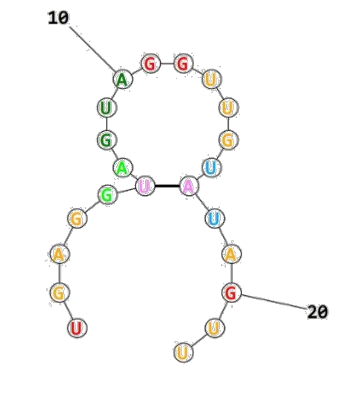

* Sequences were retrieved from the miRBase database (www.mirbase.org).

** Secondary structures were predicted using the MaxExpect algorithm within RNAstructure software (rna.urmc.rochester.edu/RNAstructure.html).
